# Chicago Medical Society, June 15, 1874

**Published:** 1874-07-01

**Authors:** Will. T. Montgomery


### CHICAGO MEDICAL SOCIETY.

### REGULAR SEMI-MONTHLY MEETING JUNE 15, 1874.

*Reported by Will. T. Montgomery.*

THE regular semi-monthly meeting of the Society was held in the parlor of the Gault House, the President, Dr. Quine, in the chair. Special order of business—Report of Section on Materia Medica.

Dr. Millard read a paper on "Ergot as a Therapeutic Agent." In his paper, the Dr. said: Although the chief uses of this remedy are confined to those affections of the uterus, over which it seems to exercise a specific action, he had been induced to give it extensive trial in other complaints, and had found most satisfactory results from its use. In parturition, he said the action of ergot was familiar to all. The constant pains it produces are characteristic, and different from natural labor pains. He thinks it acts through the ganglionic nervous system, producing a contraction of the muscular coats of the arteries. Dr. Sequard has found that ergot produces a contraction of the blood vessels of the pia mater of the dog, and that the reflex action of the spinal cord is diminished. In many of the labors he had attended, he had given ergot with the most salutary results. He had never observed any ill effects from it, to either mother or child, when judiciously given. He never gave it unless the os was dilated or dilatable. In Menorrhagia from obstructive cardiac disease, that associated with a diseased portal system, and that consequent upon a scorbutic state of the system, accompanied by an increase of the catamenia, he had found much benefit from the fresh infusion of ergot and borax.

Amenorrhœa occurring in the plethoric is best treated for a few days with salines, aloetic purgatives, or medicines which act upon and increase the circulation in the large intestines, these to be closely followed by two-ounce doses twice a day, of the infusion of ergot.

In Hæmoptysis, in the commencement of phthisis, he had given ergot with good effect, but would not prescribe it in the advanced stages, as it then sometimes produced vomiting.

He had found great benefit from it in hæmaturia, and thought it was far superior to either turpentine or the mineral acids.

Constipation of the paralytic is remedied by this drug when the most powerful cathartics fail. He had used the infusion of ergot as an injection in gleet, with good results, three or four times daily.

In the discussion that followed, Dr. N. S. Davis said: The subject is an important one, and may be studied with benefit. It is but a few years since this drug was entirely limited to parturient cases. It is now known to have a specific action upon the ganglionic centres. Whether it has a specific action upon the vascular system except through the nervous centres, he was not then prepared to say. He began to use ergot in cases of hœmorrhage as long as fifteen years ago. He used it in combination with iron, in three cases, with very satisfactory results. A case was related in which he continued to administer it for a number of months, with decided benefit. Some eminent physicians have recommended the use of ergot in cerebro-spinal congestions. He thought it was more particularly useful in the aplastic variety of cerebro-spinal disease. It increased the contractility of the vessels of the brain, and thus lessened the congestion. He had not found it so useful in sthenic cases.

Dr. E. F. Ingals said he had recently had occasion to look up the subject of ergot, and came to the conclusion that it does not always have a specific action upon the uterus, in parturient cases.

Vice President Paoli stated, that forty years ago ergot was known, in the drug stores of Europe, as a remedy for producing abortions, but pregnant women had repeatedly eaten of the bread made of it without experiencing the specific effect. He does not believe much in its specific action, and does not think much of it as a hæmostatic. Dr. Pierson had not used it much, and does not have much faith in it. Dr. Stillians has not had much experience with it in labor cases. He used it in a case of retained placenta, and produced hour-glass contraction. Has used it in combination with iron, in uterine hæmorrhage, with good results.

Dr. Thompson thinks it always has a specific action when the drug is good. Dr. Walton is of the same opinion. Dr. Gapin was glad to know so many are losing faith in ergot, and wants to see a more vigorous stand taken against its use in labor. His idea of its action upon non-striated muscular fiber was not in accordance with his idea of natural labor pains. The one was continuous, the other intermittent. The Secretary, Dr. Hutchinson, is a friend of ergot, even if it is abused. From his own observations, he has no doubt that it produces uterine contractions. He had used it, in connection with other remedies, in hæmorrhages and cerebro-spinal troubles, when it seemed to do good. He has no doubt of its abortive powers and is careful in giving it in labor cases, but thinks if properly administered it is a useful remedy, and he should continue to use it. Dr. Etheridge being present, Dr. Earle wished to hear from him upon the action of ergotine. Dr. Etheridge said that ergotine is being extensively used hypodermically, in the treatment of uterine fibroids, and with good results. A number of cases have been reported as cured. Dr. Hildebrandt first called attention to this remedy in the treatment of uterine fibroids. It acts through the vaso-motor nerve centres, shutting off the supply of blood to the tumor. He thinks the action of ergot has been demonstrated by the experiments for the cure of epilepsy. The Dr. referred to quinine as an oxytocic. He related a case in which it seems to have this effect: A lady seven months pregnant was having chills, and he prescribed quinine in four-grain doses, and when he called next day, she had miscarried. There was no sign of it before the medicine was given.

Dr. Earle referred to a paper on Ergotine in Uterine Fibroids, by Dr. Parvin, published in the *American Practitioner*. The hypodermic injections should be made once a day, and were first made in the region of the uterus, but it is found to act as well if made in the arm.

Dr. C. M. Fitch has discontinued the use of ergot in labor cases, but has used it with apparent benefit in hæmoptysis. He has had no cases of postpartum hæmorrhage for a long time, and attributes it to the moderate use of stimulants, and close attention to keeping the extremities of his patients warm.

The President, Dr. Quine, asked a question as to the distribution of nerves to the uterus, and their physiological action? Dr. Thompson said he had observed cases of labor in which the neck of the uterus was flaccid, at the same time there was firm contraction of the body of the organ. The President further inquired if any one had had any experience with ergot in cases of threatened abortions? Dr. Fitch had used it in one case, and the abortion was prevented, and the case went on to full time.

Dr. Montgomery said he was called to a case of threatened abortion, at the end of the third month, and, on making an examination, found the membranes protruding from the os, and decided that the case was too far gone to arrest their expulsion. As there was considerable hæmorrhage, he gave ergot to hasten the expulsion of the fœtus, but it was not expelled while the patient was under observation—about a week—though a rapid recovery was made. The case was lost sight of, so that he knew not whether or no the fœtus was carried to term. Dr. Hutchinson said it was another example of how patients would sometimes recover, in spite of the Dr. and his medicines. The President had given ergot in one case of threatened abortion, and it did not arrest it. He has no doubt of the specific action of the drug upon the uterus. He thinks ergot as reliable as a parturifacient as any narcotic remedy is in its action. The dose modifies its action, small doses producing natural contractions, large doses continuous. When given in moderate doses it produces increased vascular tension. In large doses, it first increases vascular tension, but, if continued, relaxes it. He had produced dilatation of the os by large doses, but contraction by small doses. After some explanations, the discussion closed, and after miscellaneous business was disposed of, a motion to adjourn prevailed.

